# Pectoral Fin Propulsion Performance Analysis of Robotic Fish with Multiple Degrees of Freedom Based on Burst-and-Coast Swimming Behavior Stroke Ratio

**DOI:** 10.3390/biomimetics9050301

**Published:** 2024-05-18

**Authors:** Zonggang Li, Bin Li, Haoyu Li, Guangqing Xia

**Affiliations:** 1 School of Mechanical Engineering, Lanzhou Jiaotong University, Lanzhou 730070, China; libinpersonal@126.com (B.L.); lihaoyu1997@outlook.com (H.L.); 2 Robotics Institute, Lanzhou Jiaotong University, Lanzhou 730070, China; 3 State Key Laboratory of Structural Analysis for Industrial Equipment, Dalian University of Technology, Dalian 116024, China; gq.xia@dlut.edu.cn

**Keywords:** bionic robotic fish, pectoral fin, stroke ratio, computational fluid dynamics, hydrodynamics

## Abstract

The pectoral fin propulsion of a bionic robotic fish always consists of two phases: propulsion and recovery. The robotic fish moves in a burst-and-coast swimming manner. This study aims to analyze a pair of bionic robotic fish with rigid pectoral fin propulsion with three degrees of freedom and optimize the elliptical propulsion curve with the minimum recovery stroke resistance using computational fluid dynamics methods. Then, the time allocated to the propulsion and recovery phases is investigated to maximize the propulsion performance of the bionic robotic fish. The numerical simulation results show that when the time ratio of the propulsion and recovery phases is 0.5:1, the resistance during the movement of the robotic fish is effectively reduced, and the drag-reducing effect is pronounced. According to a further analysis of pressure clouds and vortex structures, the pressure difference between the upstream and downstream fins of the pectoral fin varies with different stroke ratios. The increase in recovery phase time helps to prevent premature damage to the vortex ring structure generated during the propulsion process and improves propulsion efficiency.

## 1. Introduction

The bionic underwater robot is a kind of robotic system that uses the swimming mechanism of real fish to carry out planar or spatial movements, such as fast swimming and turning underwater, which has significant application value in underwater exploration, biological monitoring, military reconnaissance, and combat due to its advantages of good maneuverability, excellent propulsive performance, and stealth nature. It has been a hot spot of domestic and foreign research in recent years.

The two major swimming modes of fish in the ocean are the body and/or caudal fin (BCF) mode and the median and/or paired fin (MPF) mode [1]. Through in-depth research on the swimming mechanism of fish [1,2,3], researchers have developed various unique biomimetic robotic fish with different swimming behaviors. In order to analyze and solve the interaction problem between the motion of robotic fish and the surrounding flow field structure, most current studies use numerical simulation methods to process the flow field to obtain physical quantities such as the vortex structure and velocity vector, thereby elucidating the swimming mechanism of robotic fish in the flow field and optimizing its behavior. Xu and Wan [4] used the overlapping grid method to place the rigid pectoral/caudal fin into the flow field and relied solely on the swing of the pectoral fin to drive the fish to swim straight and turn. ChuiJie and Liang [5] conducted research on the flexible deformation and autonomous swimming of the tail fin of a biomimetic robotic fish. The above research achieved some results in analyzing the hydrodynamic performance of a single pectoral fin’s swing or the caudal fin’s fluctuation but did not consider the synergistic effect when the pectoral and caudal fins move simultaneously.

The coordinated swimming movements of pectoral and caudal fins combines the swinging of pectoral fins and the fluctuation of caudal fins on the basis of the aforementioned literature, and through the combined action of the two, the fish body takes corresponding actions. Compared to simple pectoral or caudal fin propulsion, this coordination has obvious advantages in propulsion performance and efficiency. With the fusion of pectoral and caudal fin vortices, robot fish have better propulsion performance and maneuverability. In a study of coordinated swimming movements of pectoral and caudal fins, B et al. [6] analyzed the flexible multi-fin dynamics of pufferfish. A simulated pufferfish model containing flexible dorsal fins, anal fins, and caudal fins was inserted into the flow field for propulsion, and its hydrodynamic performance and vortex structure were analyzed. Carling et al. [7] studied the changes in swimming speed and wake vortices of a continuous eel model during autonomous propulsion.

In the study of the motion laws of the pectoral fins of biomimetic robotic fish, Martin and Gharib [8] regarded a rectangular rigid plate with conical edges as a universal pectoral fin. Using the thrust and operational efficiency monitored by force sensors during the motion process as indicators, they evaluated the trajectories of different pectoral fins, including ellipses, “8” shapes, and straight lines. Through physical experiments, they ultimately obtained a set of optimal thrust operational trajectories. Low et al. [9,10,11,12] substituted elliptical and “8”-shaped motion curves into the pectoral fin motion of biomimetic sea turtles for hydrodynamic analysis and experiments. Gibb et al., Lauder and Jayne, and Westneat and Walker [13,14,15] conducted experiments and simulations using the “8”-shaped motion curve as the pectoral fin motion law of biomimetic fish.

In other research related to biomimetic robotic fish, Zheng et al. [16] established an artificial lateral line system composed of pressure sensor arrays to sense the changes in the surrounding flow field when the robotic fish swims. This study verified the effectiveness and practicality of the artificial side-line system. In addition, Yu et al. [17] studied the steering control problem of multi-link robotic fish, developed a method to control the turning gait of robotic fish, and verified the applicability of this method through simulation and physical experiments. Li et al. [18] used numerical simulation to study the intermittent propulsion performance of fish tail fins, and the results showed that adding a sliding stage in continuous propulsion can effectively improve the swimming efficiency of fish, and there is an optimal propulsion gait.

Based on the computational fluid dynamics (CFD) method, the effect of the stroke ratio of the pectoral fin oscillation curve on the swimming speed and hydrodynamic coefficient of the robotic fish was numerically simulated. In this paper, the robotic fish generates self-propulsion in the flow field through pectoral fin oscillations. The pectoral fins adopt an elliptical oscillation curve. The robotic fish’s caudal fins are kept fixed to eliminate the interference of tail fin fluctuations with pectoral fin movement. The pectoral fin propulsion phase time is fixed at 0.5 s, and the time spent in the pectoral fin recovery phase is changed to change the pectoral fin motion stroke ratio. This optimizes the stroke ratio of the pectoral fin swing curve to improve the swimming efficiency and stability of the robotic fish.

## 2. CFD Modeling of Robot Fish

### 2.1. Three-Dimensional Robot Fish Model and Related Dimensions

**Figure 1:**
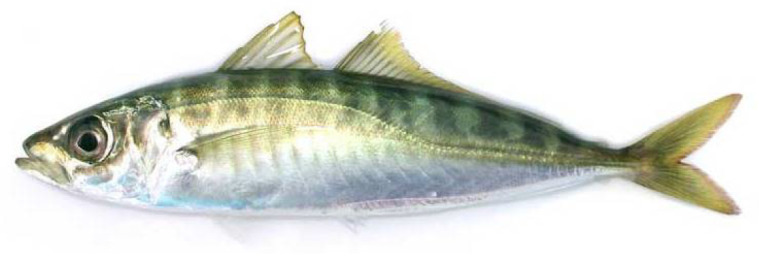
Deep-bodied round scad.

**Figure 2:**
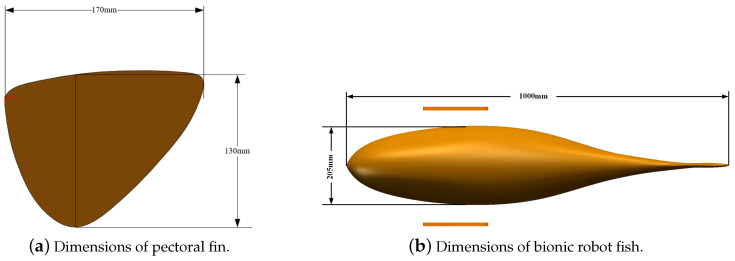
Shape and size of bionic robotic fish.

**Figure 3:**
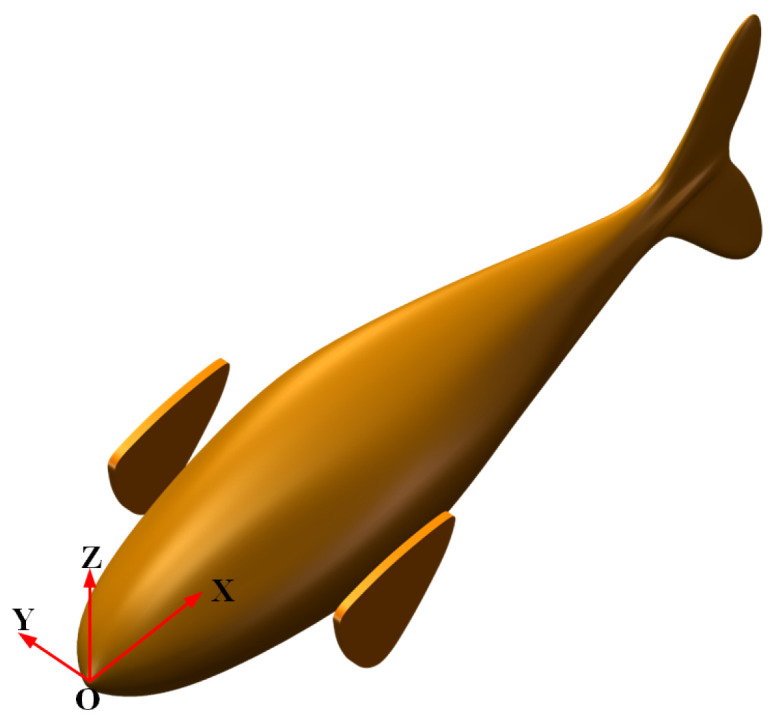
Robot fish with coordinate system.

We used the deep-bodied round scad as a bionic object, as shown in Figure 1. Then, the shape was simplified and enlarged, and a three-dimensional (3D) geometric model of the bionic robotic fish was designed, as shown in Figure 2. The pectoral fins and the body of the bionic robotic fish adopt separate structures, where the overall length of the robotic fish is 1000 mm, the width is 205 mm, and the height of the caudal fin is 370 mm. The model of the bionic pectoral fin has a spread length of 170 mm and a maximum chord length of 130 mm. The robotic fish coordinate system is shown in Figure 3.

### 2.2. Overview of Pectoral Fin Swing Curves and Stroke Ratios

The pectoral fins are coupled with three degrees of freedom, namely, rowing, flapping, and feathering motions, to realize the desired trajectory, in which the one-to-one correspondence between the flapping and rowing of the pectoral fins, as well as the change in the rocking wing angle over time in one pectoral fin oscillation cycle, is called the pectoral fin oscillation curve [19]. According to the principle of bionics, that is, the swinging pattern of the pectoral fins of aquatic organisms in nature, the pectoral fin swing curve can be roughly divided into two patterns: the elliptical swing curve and the “8” swing curve. The following equation gives the kinematic model of the elliptical swing curve:

(1) $$\left\{\begin{array}{c} \Phi_{R}=\phi_{RC}-\phi_{RA}\cos\left(\omega t\right)\\ \Phi_{F}=\phi_{FC}-\phi_{FA}\cos\left(\omega t+\Delta_{F}\right)\\ \Phi_{FL}=\phi_{FLC}-\phi_{FLA}\cos\left(\omega t+\Delta_{FL}\right) \end{array}\right.$$

where $\Phi_{R}$, $\Phi_{FL}$, and $\Phi_{F}$ represent the Euler angles of the pectoral fin’s rowing, flapping, and feathering motions, respectively; $\phi_{RC}$, $\phi_{FC}$, and $\phi_{FLC}$ represent the initial phases of the rowing, flapping, and feathering motion angles; $\phi_{RA}$, $\phi_{FA}$, and $\phi_{FLA}$ represent the amplitudes of the three motions, respectively; $\Delta_{F}$ and $\Delta_{FL}$ represent the phase differences; ω=2π/T is the angular frequency of the motions; *T* is the period; and *t* is the time.

**Figure 4:**
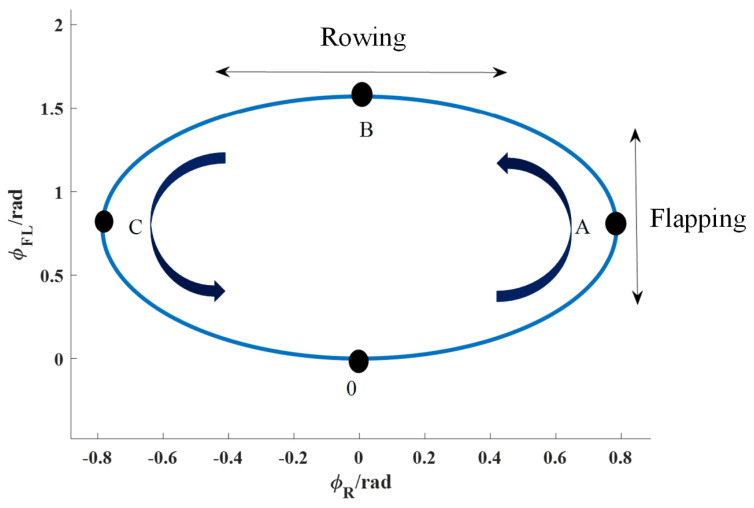
A schematic diagram of the movement curves of pectoral fins rowing and flapping.

Figure 4 shows a schematic diagram of the elliptic-type curve of the pectoral fin in one cycle, where the arrows represent the direction of the pectoral fin motion, the initial position of the pectoral fin is point O, and the initial position of the rocker angle is in the vertical direction, which is stipulated to initially be 0 degrees. In the direction facing the Y-axis, clockwise rotation is stipulated to be in the negative direction, and anticlockwise is in the positive direction, and in the elliptic-type rocking curve, the pectoral fin moves sequentially to points A, B, C, and O in a single rocking cycle from point O along its rocking curve.

**Figure 5:**
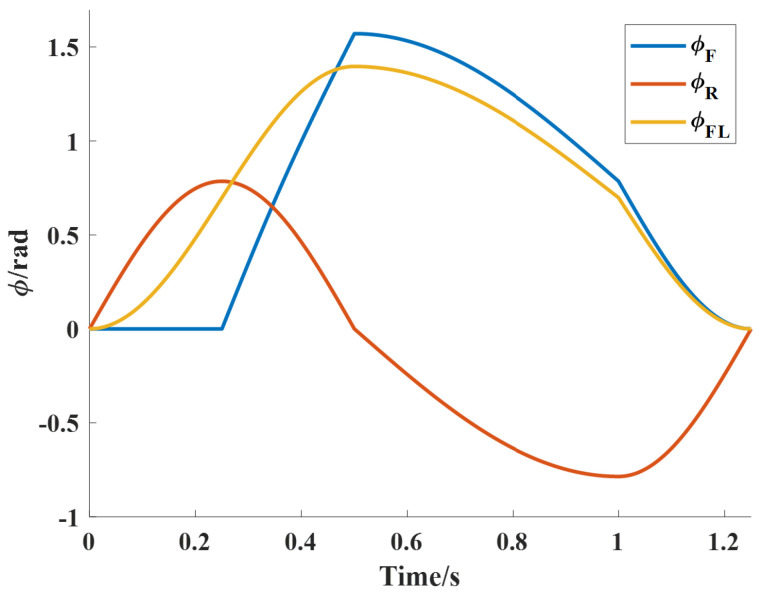
Changes in the flapping, rowing, and feathering angles of the pectoral fin over time after improvement.

In this paper, the operation of the pectoral fin adopts an optimized elliptical swing curve, and its rowing angle $\Phi_{R}$, flapping angle $\Phi_{FL}$, and feathering $\Phi_{F}$ angle over time are shown in Figure 5. The phase in which the front and rear beat angles of the pectoral fin increase with time in the unit cycle is defined as the propulsion stroke, and the phase in which the front and rear beat angles decrease with time is defined as the recovery stroke. In Figure 5, it can be seen that the pectoral fins in the first cycle are in the phase of propulsion at 0–0.25 s versus 1–1.25 s, and the pectoral fins are in the phase of recovery at 0.25–1 s.

The “burst-and-coast” swimming mode is a common swimming mode for fish and other aquatic organisms. Viderer et al. [20,21] conducted the theoretical modeling and experimental analysis of the different swimming modes of Cod. Their research findings indicate that the “burst-and-coast” swimming mode consumes less energy at high speeds. Blake et al. [22] found that fish with slender body size ratios between 4.0 and 6.5 were most advantageous for “burst-and-coast” swimming mode. Li et al. [18] analyzed the “burst-and-coast” swimming mode of caudal fins of undulating fish. They found that adding an unpowered coast phase between continuous propulsion phases could improve the swimming efficiency of robotic fish and reduce energy consumption. In nature, the oscillation of the pectoral fins always makes the fish body show a certain “burst-and-coast” behavior; i.e., in addition to the acceleration phase, in which the pectoral fins flap backward to push the fish body with the help of the force of the water, the pectoral fins also undergo a process of swinging back and entering relative stasis between two oscillation phases. That is to say, the process of pectoral fin propulsion comprises not only a propulsion phase that generates thrust but also a recovery phase and a stationary coast phase. In both the recovery and stationary phases, there is no thrust generated, and the fish body coasts forward through inertia. The above two phases are called the coast phase of the robotic fish. Therefore, in the study of the pectoral fin oscillation law, the “burst-and-coast” model is also applicable. In this paper, we focus on the effect of the time ratio of the pectoral fin recovery phase per unit cycle in the coast phase on the swimming performance and swimming efficiency of robotic fish.

The stroke ratio is the ratio of the time taken by the pectoral fin in the propulsion phase and the recovery phase in one cycle. The pectoral fin movement stroke ratio was changed by changing the recovery phase movement time under the condition that the propulsion phase time was the same, and the different stroke ratios of the pectoral fin in one unit cycle are shown in the schematic diagram in Figure 5.

**Figure 6:**
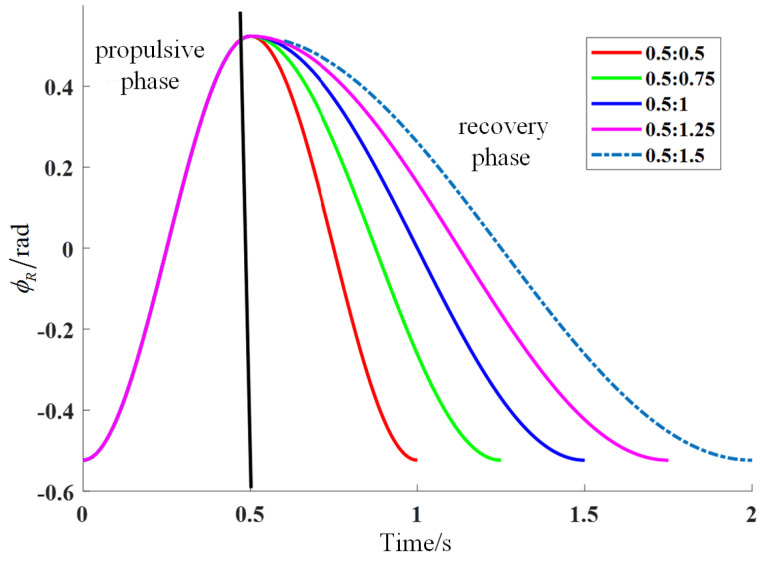
Schematic diagram of pectoral fin recovery stroke ratio.

As can be seen in Figure 6, five stroke ratios of 0.5:0.5, 0.5:0.75, 0.5:1, 0.5:1.25, and 0.5:1.5 were set for the pectoral fins in this paper, and the pectoral fin propulsion phase was consistent at each stroke ratio. The time of the recovery phase increased with the decrease in the stroke ratio.

### 2.3. Mesh Partitioning Case and Convergence Validation

**Figure 7:**
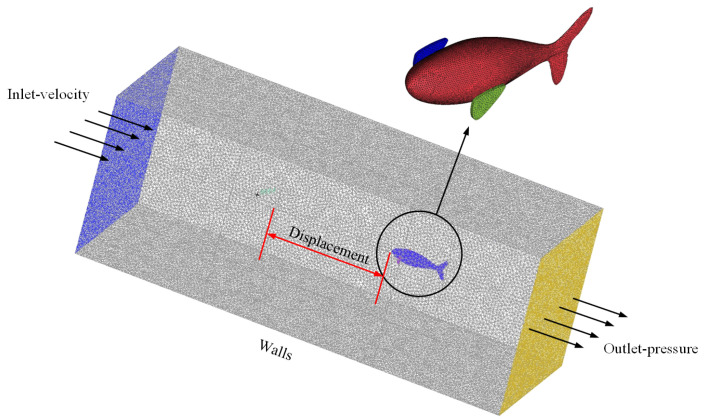
Flow field grid division.

The mesh division for numerical simulations is shown in Figure 7. The model needs to self-propel to swim forward. Let the body length of the robot fish be L. The dimension of the computational domain is 8 L × 3 L × 3 L, and the mesh in the X-direction is uniformly distributed in order to ensure that the computation around the fish always maintains the same accuracy during the fish’s swimming process. The red line indicates the displacement of the robotic fish moving forward in the flow field. The computational area was divided by tetrahedral unstructured meshing, and the walls of the model were meshed with triangles. The meshes of the pectoral fins, caudal fins, and fish body parts were locally encrypted to ensure computational accuracy. In this study, a dynamic mesh technique based on the elastic smooth method and local reconstruction method was used to ensure the mesh quality in the fluid domain during the pectoral fin movement. The mesh quality of the robotic fish was kept above 0.3 to satisfy the required quality for the computation, and all subsequent analyses were based on the mesh shown in Figure 7.

In the numerical simulation, in order to verify the convergence of the grids, three grid sizes were taken, and the numerical simulation of the same condition was carried out for each grid; finally, the hydrodynamic coefficients of each grid were calculated, as shown in Figure 7, in which grids 1, 2, and 3 correspond to the sizes Coarse, Medium, and Fine, respectively.

**Figure 8:**
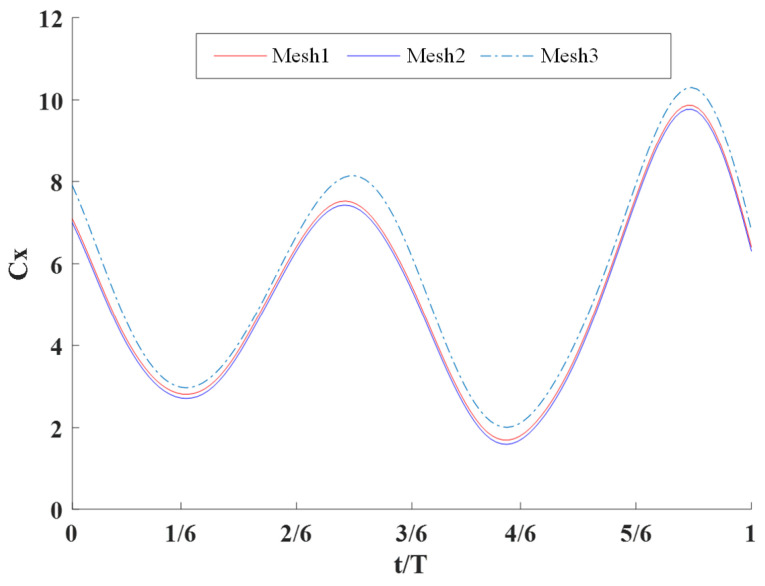
Verification of grid convergence.

In Figure 8, it can be seen that this study set a total of five stroke ratios, 0.5:0.5, 0.5:0.75, 0.5:1, 0.5:1.25, and 0.5:1.5, for the rowing motion pattern of the pectoral fin. At each stroke ratio, the pectoral fin burst phase remains the same, and the recovery phase time gradually increases as the stroke ratio decreases.

As can be seen in the figure, the calculation results using the three meshes are basically the same, so the mesh setting and numerical simulation method are reasonable and convergent, and the subsequent simulation results were all obtained by mesh 2 calculations.

### 2.4. Additional Parameters in the Flow Field

The pectoral fin thrust coefficient and lift coefficient of the robotic fish during oscillation can be expressed by the following equations, respectively:

(2) $$C_{x}=\frac{2F_{x}}{\rho U^{2}S},C_{z}=\frac{2F_{y}}{\rho U^{2}S}$$

where ρ is the fluid density, $F_{x}$ and $F_{z}$ are the thrust and lift forces on the robot fish, respectively, and *S* is the projected area of the fish.

## 3. Control Equations and Numerical Simulation of Flow Fields

The simulation of underwater robots usually does not consider heat exchange, so the energy conservation equation is generally not considered, and the basic control equations of the flow field are the continuity equation and the N-S equation. The equations controlling the robotic fish bypassing flow problem are the viscous incompressible N-S equations with the following expressions:

(3) $$\left\{\begin{array}{c} \nabla \cdot u=0\\ \frac{\partial u}{\partial t}+\left(u\cdot \nabla \right)u=-\frac{1}{\rho }\nabla p+\mu \nabla^{2}u \end{array}\right.$$

where *u* is the velocity of the fluid, *p* is the pressure of the fluid, and ρ is the fluid density.

The computational region conditions are as follows: The velocity and pressure gradients at the inlet and outlet boundaries are 0. Since higher accuracy is required for the near-wall flow field, the turbulence model adopted in this paper is the SST k−ω model with low-Re-number calibration. The SIMPLE algorithm is used to couple the pressure and velocity in the continuous equations, where the pressure, momentum, and turbulent kinetic energy are in the second-order windward format. The hydrodynamic coefficients of the fish body in three directions are monitored, along with the velocity and displacement in the x-direction.

We use the dynamic mesh technique to realize the motion of the pectoral fin and fish body. We use spring approximation mesh smoothing and local mesh reconstruction methods. This method can prevent the negative volume phenomenon of the grid caused by the volume being less than zero during the deformation and reconstruction of grid cells.

## 4. Numerical Simulation and Analysis of Results

### 4.1. Influence of Stroke Ratio on Hydrodynamic Characteristics of Robotic Fish

**Figure 9:**
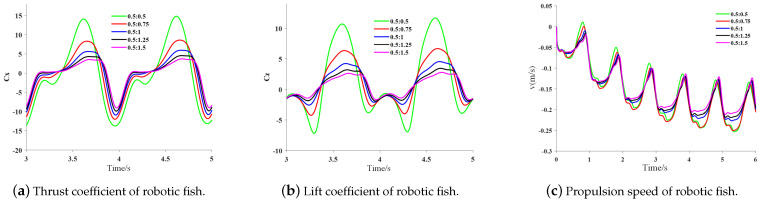
The variation in the hydrodynamic coefficients and velocity over time with different stroke ratios.

Through the simulation calculation of different stroke ratios, the thrust coefficient and lift coefficient of the robotic fish in the third and fourth cycles in the stable cruising state and the propulsive speed of the robotic fish in all cycles are obtained, as shown in Figure 9. With the change in time spent in the recovery phase, the corresponding stroke ratio becomes smaller. In the thrust coefficient curve, a negative longitudinal axis means that as the proportion of time spent in the propulsive phase of the pectoral fins and the recovery phase changes with the stroke ratio, the robot fish is subjected to thrust for a longer period. A positive longitudinal axis means that the robot fish is obstructed, so in the velocity curve, the negative direction means that the robot fish swims forward; in the lift coefficient curve, a longitudinal axis value of 0 or above is the lift, and 0 or below is a lower potential.

**Figure 10:**
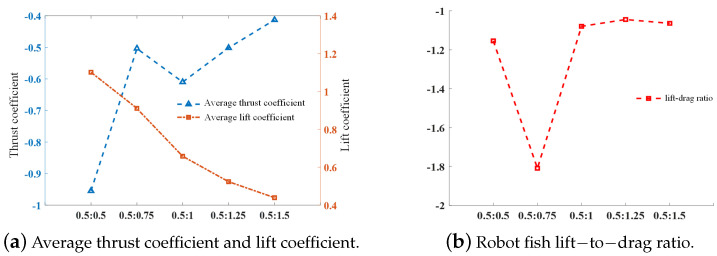
Average thrust coefficient, lift coefficient, and lift drag ratio at different stroke ratios.

As can be seen in Figure 9a, as the proportion of time spent in the recovery phase increases, the end of the first half of the pectoral fin propulsion phase of the robotic fish is gradually advanced, and the end of the pectoral fin propulsion phase occurs earlier when the stroke ratios are 0.5:1.25 and 0.5:1.5; the pectoral fins produce thrust in the first half of the recovery phase, and the larger the stroke ratio, the larger the thrust it produces. Stroke ratios of 0.5:1.25 and 0.5:1.5 produce essentially no thrust during this phase, and larger stroke ratios result in an increase in the frequency of pectoral fin motion during the recovery phase, thus producing thrust fluctuations during this phase; the peak thrust coefficient of the pectoral fins decreases with decreasing stroke ratios around the second half of the cycle, and the greatest thrust and drag are produced at a stroke ratio of 0.5:0.5, whereas essentially no thrust or drag is produced at stroke ratios of 0.5:1.25 and onward, and the thrust loss is small. In Figure 9b, it can be seen that the lift coefficient of the robotic fish shows large fluctuations with the alternation of the two phases of the pectoral fin, and the fluctuation of the lift coefficient gradually increases with the increase in the stroke ratio.As the stroke ratio decreases, the fish is subjected to a lower potential as it gradually decreases. The robotic fish is subjected to a certain amount of lift in the second half of the cycle, and as the pectoral fin stroke ratio decreases, it enters the propulsion phase in sequence and begins to be subjected to a lower potential. In Figure 9c, it can be seen that at all stroke ratios, the fish enters a stable cruising state after four cycles, and the peak cruising speed of the fish is the largest when the stroke ratio is 0.5:0.75. With the decrease in the stroke ratio, the pectoral fin period gradually increases due to its constant propulsive phase, which leads to the increasing speed loss of the fish, but its fluctuation is relatively smooth, and the fish has good stability in the flow field. The influence of the stroke ratio on the average thrust, average lift, and lift-to-drag ratio during the swimming process of the robotic fish is shown in Figure 10.

As can be seen in Figure 10a, the average thrust coefficient during the movement of the robotic fish shows a decreasing trend with the increase in the time of the pectoral fin recovery phase. When the stroke ratio is 0.5:0.75, the pectoral fin recovery phase still produces a large resistance, which hinders the swimming of the robotic fish, while the pectoral fins have a better drag-reducing effect at 0.5:1, and the two produce comparable thrust in the propulsive phase. The average thrust coefficient of the former is smaller than that of the latter. The average thrust coefficient is the largest when the stroke ratio is 0.5:0.5, but the fluctuation of the thrust coefficient curve throughout the whole cycle is also the largest, according to the previous analysis, so the stability of the robotic fish with this pectoral fin oscillation curve is not good during the swimming process. The robotic fish with the oscillation curve with a stroke ratio of 0.5:1 has good stability with not much loss in thrust. With the decrease in the stroke ratio, the robotic fish has a good stability, and as the stroke ratio decreases, the average lift coefficient of the fish also tends to decrease, but regardless of the swing curve, the pectoral fins provide a certain amount of lift to the fish in general. In Figure 10b, it can be seen that the lift-to-drag ratio of the fish is the smallest when the stroke ratio is 0.5:0.75, and the lift-to-drag ratio does not change much when the other four swing curves are used.

### 4.2. Effect of Stroke Ratio on Swimming Performance and Swimming Efficiency of Robotic Fish

The swimming performance and mechanism of the robotic fish in the flow field at different stroke ratios are analyzed by velocity vectors, pressure maps, and three-dimensional vortex structures. The stroke ratio of the pectoral fin recovery is optimized to improve the efficiency of the robotic fish through the characteristics of the flow field.

#### 4.2.1. Analysis Using Velocity Vectors and Pressure Cloud Maps

**Figure 11:**
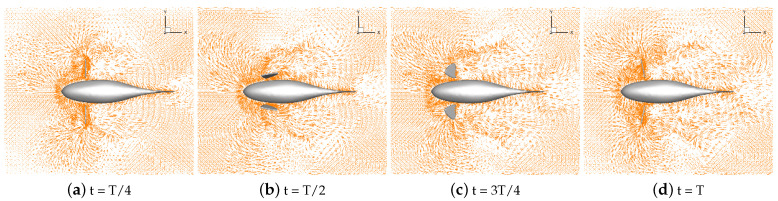
Velocity vectors in the flow field at a stroke ratio of 0.5:0.5.

**Figure 12:**
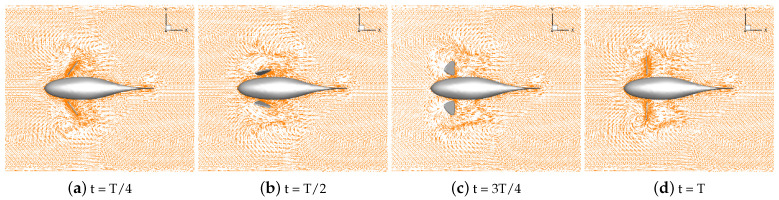
Velocity vectors in the flow field at a stroke ratio of 0.5:0.75.

**Figure 13:**
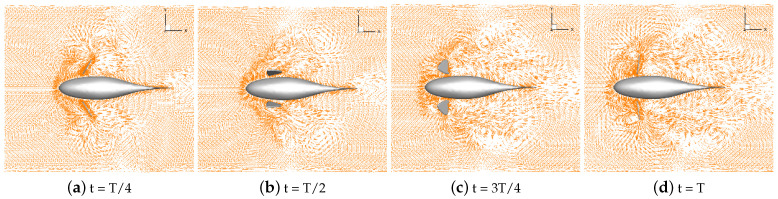
Velocity vectors in the flow field at a stroke ratio of 0.5:1.

**Figure 14:**
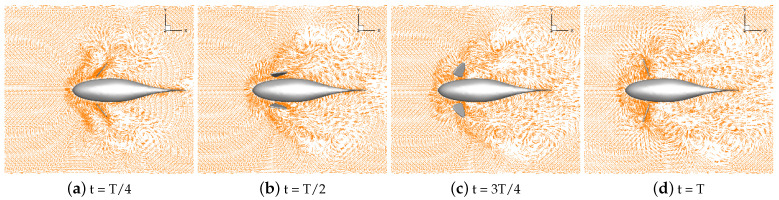
Velocity vectors in the flow field at a stroke ratio of 0.5:1.25.

**Figure 15:**
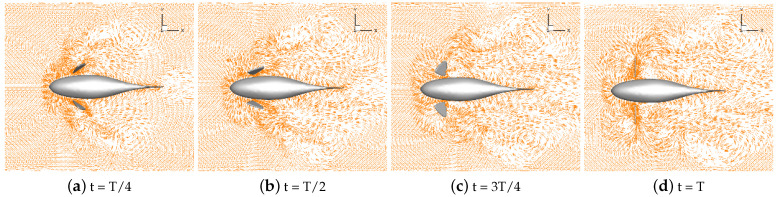
Velocity vectors in the flow field at a stroke ratio of 0.5:1.5.

Numerical simulations of self-propulsion in static water were carried out separately for robotic fish with pectoral fin swing curves with different stroke ratios. The caudal fin was kept fixed, and the bionic robotic fish achieved its swimming behavior in the flow field only through the swing of its pectoral fins. The velocity vectors of the flow field around the fish at eight typical moments of the third cycle at each stroke ratio are shown in Figure 11, Figure 12, Figure 13, Figure 14 and Figure 15.

As can be seen in Figure 11, Figure 12, Figure 13, Figure 14 and Figure 15, since the behavior and duration of the pectoral fin propulsion phase at different stroke ratios are unchanged, the magnitude and direction of the velocity around the robotic fish are basically the same at the same moment in this phase. The pectoral fins oscillate backward during this phase and generate a vortex that is conducive to the forward swimming of the robotic fish. This vortex will gradually move backward with the robotic fish and detach itself from the body of the fish, and the thrust force exerted by the vortex on the body of the fish in the process gradually decreases. The thrust force acting on the fish body in the propulsion phase does not change with the change in the stroke ratio. In the early phase of the pectoral fin recovery phase, i.e., t = 2T/8 to 4T/8, the pectoral fins with smaller stroke ratios oscillate with a low frequency, which results in a smaller velocity vector around the pectoral fins and decreases the interference effect on the backward-moving vortex, so the smaller the stroke ratio is, the lower the resistance force acting on the pectoral fins is. Later in the recovery phase, i.e., t = 4T/8 to 7T/8, the distance between the vortex and the fish body gradually increases with the decrease in the stroke ratio. The spacing between the front and back vortices also gradually increases, so the resistance of the robotic fish in the late phase also decreases with the decrease in the stroke ratio.

**Figure 16:**
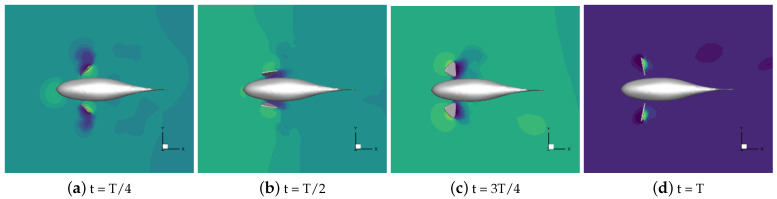
A pressure nephogram in the flow field at a stroke ratio of 0.5:0.5.

**Figure 17:**
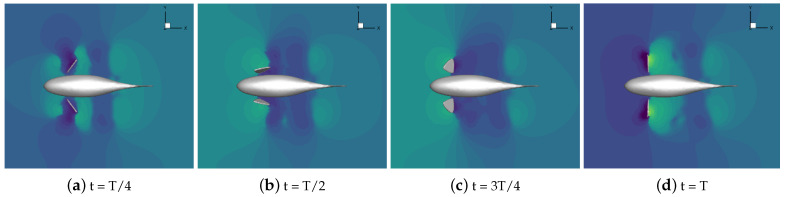
A pressure nephogram in the flow field at a stroke ratio of 0.5:0.75.

**Figure 18:**
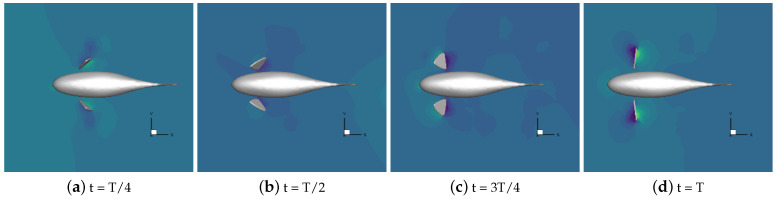
A pressure nephogram in the flow field at a stroke ratio of 0.5:1.

**Figure 19:**
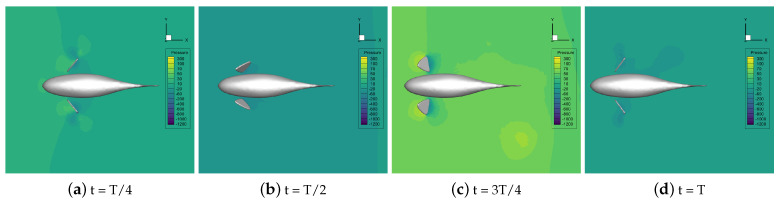
A pressure nephogram in the flow field at a stroke ratio of 0.5:1.25.

**Figure 20:**
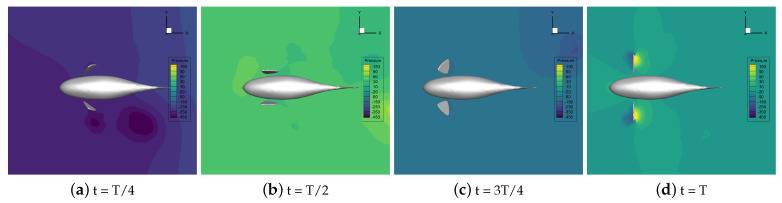
A pressure nephogram in the flow field at a stroke ratio of 0.5:1.5.

The velocity vector of the flow field during the self-propelled motion of the robotic fish was analyzed at different stroke ratios. The results indicate that the distance between the vortices generated simultaneously by the pectoral fin swing and the fish body gradually increases as the stroke ratio decreases. The increase in the duration of the recovery phase effectively prevents the vortex generated by the pectoral fins in the propulsion phase from being destroyed in advance and gives full play to the propulsion effect of the vortex on the robotic fish. Decreasing the oscillation frequency of the pectoral fins in the recovery phase can reduce the resistance of the fish. The horizontal pressure distribution of the flow field around the fish with different stroke ratios is shown in Figure 16, Figure 17, Figure 18, Figure 19 and Figure 20.

In Figure 16, Figure 17, Figure 18, Figure 19 and Figure 20, it can be seen that, in the propulsion phase, i.e., from t = T/8 to t = 2T/8, the pressure difference between the areas upstream and downstream of the pectoral fins is obvious. The downstream pressure of the pectoral fins is larger and generates a high-pressure vortex downstream, and the larger pressure difference provides the thrust for the robotic fish. The pressure distribution around the pectoral fins in this phase does not change with the change in the stroke ratio. In the pre-recovery phase, i.e., the period from t = 2T/8 to t = 4T/8, the pectoral fins move upward, the pressure difference is smaller, and the robotic fish is subjected to lower resistance. By coasting forward, due to the smaller stroke ratio, the pectoral fins move more slowly, resulting in a further decrease in the differential pressure. At a stroke ratio of 0.5:1.5, the downstream pressure difference on the fin surface is basically 0. In the late recovery phase, i.e., from t = 4T/8 to 7T/8, the pectoral fins move downward and produce differential pressure in the opposite direction, and the differential pressure decreases with the reduction in the stroke ratio, so the robotic fish at this time is subject to the drag force. This pressure difference decreases with the decreasing stroke ratio, so the resistance of the robotic fish decreases with the decreasing stroke ratio. At t = 7T/8 to t = T, the pectoral fins revert to the propulsive phase and generate a high-pressure vortex downstream to push the robotic fish to swim.

Through the analysis of the pressure distribution of the flow field in the horizontal plane around the robotic fish at different stroke ratios, it can be seen that the drag-reducing effect of the stroke ratio is mainly reflected in the whole recovery phase of the pectoral fin. The different stroke ratios lead to differences in the movement behavior and movement frequency of the pectoral fin in the phase, which, in turn, affects the size of the downstream pressure difference on the pectoral fin surface. The time share of the pectoral fin’s propulsion phase and recovery phase can be optimized through a comparison of the pressure difference situations. The larger the stroke ratio, the larger the proportion of time in the propulsion phase and the larger the thrust of the fish in the swimming phase. But at the same time, the recovery phase will also have a large reverse pressure difference, hindering the coasting movement of the fish. The smaller the stroke ratio, the more obvious the effect of drag reduction, but if the pectoral fin spends too little time in the propulsion phase, the thrust will be insufficient. From the analysis of the pressure cloud diagrams, the pectoral fin stroke ratio is 0.5:1; that is, the time of the propulsion phase is 1/2 the time of the recovery phase.

#### 4.2.2. Analyzing the Three-Dimensional Vortex Structure

**Figure 21:**
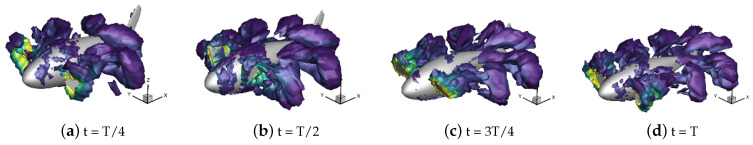
The vortex structure in the flow field at a stroke ratio of 0.5:1.

The distribution of vortex structures in the flow field around the robotic fish swimming at different stroke ratios is shown in Figure 21. The identification of 3D vortex structures is based on the Q criterion, and the color of the vortex structure reflects the size of the vorticity.

In Figure 21, it can be seen that the vortex structure is generated on both sides of the robotic fish body during its movement at all stroke ratios except for 0.5:0.5, and during the propulsion phase, the pectoral fins swing backward to generate vortex rings in the backward direction. At a stroke ratio of 0.5:0.5, in the recovery phase, pectoral fins move at the fastest frequency. The forward swing at the same time generates vortex rings moving positively toward the Z-axis, increasing the lift of the robotic fish movement. The vortex rings in the propulsion phase are under the curve with respect to the rest of the stroke ratios, so there exists a large lift in this phase. A stroke ratio of 0.5:0.75 has a more complete vortex ring with a more complete structure that only moves backward, which has a better effect in reducing the drag. Stroke ratios of 0.5:0.75, 0.75, and 0.75 have a better drag-reducing effect. At stroke ratios of 0.5:0.75, 0.5:1, and 0.5:1.25, with the stroke ratio decreasing, the interval between the vortex rings generated in the propulsion phase of the different cycles gradually increases, but the robotic fish’s coasting time is too long, and the speed loss is greater.

### 4.3. Experiment

**Figure 22:**
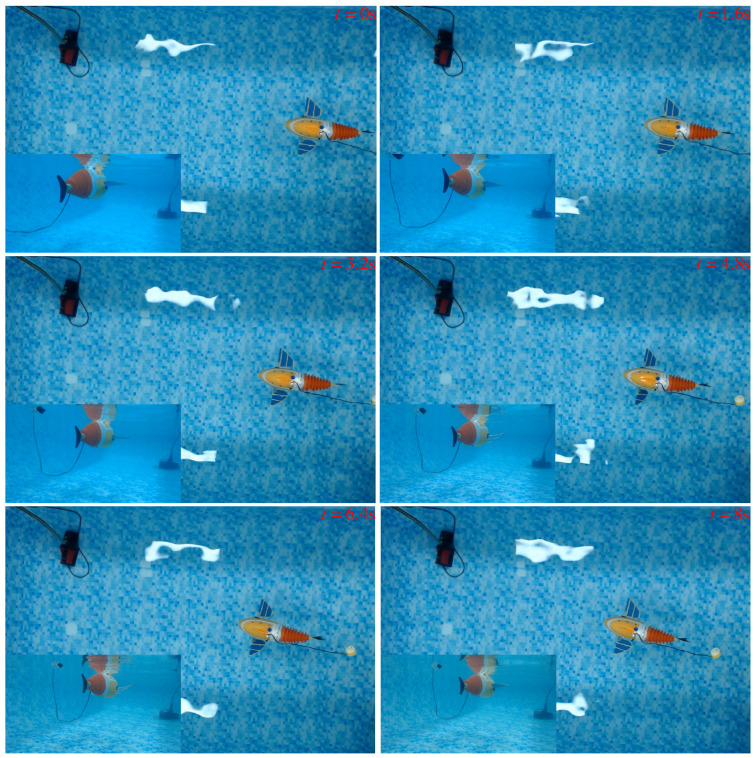
The swimming motion sequence of the robotic fish.

In order to verify the accuracy of the numerical calculations, direct swimming experiments were conducted on existing robotic fish in still water in the laboratory. And ultrasonic sensors and global cameras were used to monitor the real-time motion data of the robotic fish in the forward direction. The experiment lasted for 8 s, with the position and motion trajectory of the robotic fish at six time points shown in Figure 22.

As shown in Figure 22, as the robotic fish moves forward in the flow field, it experiences a small lateral displacement due to the periodic fluctuations of its tail fin but, overall, exhibits straight swimming behavior. The comparison between the average speed of the robotic fish in the simulation environment and the average speed in the experimental environment at different travel ratios is shown in Table 1.

From Table 1, it can be seen that the actual displacement of the robotic fish is smaller than in the simulation results, and the simulated average speed is slightly higher than the experimental average speed at different stroke ratios. The main reason is that the hydrophilic material of the robotic fish shell in the experiment, the wall effect during the experiment, and the interference of external factors increase the swimming resistance; the shell of the robotic fish can be filled with hydrophobic materials, flow field dimensions can be increased, and experimental procedures can be optimized to reduce drag and improve experimental methods. The overall error of the experiment is within the expected range, and the overall swimming trend is consistent.

## 5. Conclusions

In this paper, the ratio of the pectoral fin propulsion stroke and recovery stroke per unit propulsion cycle is used as a metric of pectoral fin propulsion efficiency, and the swimming–sliding behaviors of a robotic fish at different ratios are analyzed. The optimal ratio for the straight-line swimming of the robotic fish is obtained by analyzing the tail vortex structure to make full use of the thrust generated by the pectoral fin recovery to effectively improve the propulsive efficiency. The following conclusions were drawn:

(1) In the process of pectoral fin propulsion, a stroke ratio of 0.5:1 can effectively reduce the resistance of the fish in the process of swimming without any significant change in the amplitude of the thrust force.

(2) Increasing the duration of the recovery phase can effectively prevent the vortex generated by the pectoral fins in the propulsion phase from being destroyed in advance, giving full play to the propulsive effect of the vortex on the robotic fish, and reducing the oscillation frequency of the pectoral fins in the recovery phase also reduces the drag force on the robotic fish during swimming.

(3) The larger the stroke ratio is, the larger the reverse pressure difference is in the recovery phase of the pectoral fins, which hinders the coasting movement of the fish. The smaller the stroke ratio is, the more obvious the drag-reducing effect is, but if the pectoral fins spend too little time in the propulsion phase, there will be insufficient thrust. From the analysis of pressure cloud diagrams, the pectoral fin stroke ratio is 0.5:1; i.e., the time of the propulsion phase is 1/2 of the time of the recovery phase when the propulsion of the fish is more desirable.

(4) The increase in the duration of the recovery phase of the pectoral fins prevented the vortex and the vortex ring structure generated in the propulsion phase from being destroyed prematurely, which improved the drag reduction performance and swimming efficiency of the robotic fish during the swimming process.

## Figures and Tables

**Table 1:** Comparison of average swimming speeds of robotic fish.

Stroke Ratio	Simulated Average Speed (m/s)	Experimental Average Speed (m/s)
0.5:0.5	0.1490	0.1350
0.5:0.75	0.1697	0.1605
0.5:1	0.1480	0.1420
0.5:1.25	0.1460	0.1420
0.5:1.5	0.1421	0.1393

## Data Availability

No new data were created or analyzed in this study. Data sharing is not applicable to this article.
